# Nitric Oxide Synthase Inhibitor Attenuates the Effects of Repeated Restraint Stress on Synaptic Transmission in the Paraventricular Nucleus of the Rat Hypothalamus

**DOI:** 10.3389/fncel.2017.00127

**Published:** 2017-05-03

**Authors:** Magdalena Kusek, Anna Tokarska, Marcin Siwiec, Anna Gadek-Michalska, Bernadeta Szewczyk, Grzegorz Hess, Krzysztof Tokarski

**Affiliations:** ^1^Department of Physiology, Institute of Pharmacology, Polish Academy of SciencesKraków, Poland; ^2^Institute of Zoology and Biomedical Research, Jagiellonian UniversityKraków, Poland; ^3^Department of Neurobiology, Institute of Pharmacology, Polish Academy of SciencesKraków, Poland

**Keywords:** mIPSC, mEPSC, GABAergic, glutamatergic, corticosterone, PVN, HPA, stress

## Abstract

Corticotropin-releasing hormone (CRH)-synthesizing parvocellular neuroendocrine cells (PNCs) of the hypothalamic paraventricular nucleus (PVN) play a key role in the activation of the hypothalamic-pituitary-adrenocortical (HPA) axis. Several studies have demonstrated that synaptic inputs to these cells may undergo stress-related enhancement but, on the other hand, it has been reported that exposition to the same stressor for prolonged time periods may induce a progressive reduction in the response of the HPA axis to homotypic stressors. In the present study rats were subjected to 10 min restraint sessions, repeated twice daily for 3 or 7 days. Miniature excitatory and inhibitory postsynaptic currents (mEPSCs and mIPSCs) were then recorded from PNCs in *ex vivo* hypothalamic slice preparations obtained 24 h after the last restraint. Restraint stress repeated over 3 days resulted in increased mean frequency and decreased rise time and decay time constant of mEPSCs, accompanied by a decrease in the excitability of PNCs, however, no such changes were evident in slices obtained from rats subjected to restraint over 7 days. There were no changes in mIPSCs after repeated restraint. Administration of the unspecific nitric oxide synthase (NOS) blocker Nω-Nitro-L-arginine (L-NNA) before each restraint, repeated over 3 days, prevented the occurrence of an increase in mEPSC frequency. However, animals receiving L-NNA and subjected to repeated restraint had similar changes in PNCs membrane excitability and mEPSC kinetics as stressed rats not receiving L-NNA. Comparison of the effects of a single 10 min restraint session followed by either an immediate or delayed (24 h) decapitation revealed an increase in the mean mEPSC frequency and a decrease in the mean mIPSC frequency in slices prepared immediately after restraint, with no apparent effects when slice preparation was delayed by 24 h. These results demonstrate that restraint, lasting 10 min and repeated twice daily for 3 days, induces a selective and long-lasting enhancement of excitatory synaptic input onto PNCs, partially by a NOS-dependent mechanism, and reduces PNC excitability, whereas prolongation of repeated stress for up to 7 days results in an adaptation.

## Introduction

The paraventricular nucleus (PVN) of the hypothalamus generates an integrated physiological stress response via the activation of the hypothalamus-pituitary-adrenal (HPA) axis. A key element of the HPA axis is the population of parvocellular neurosecretory cells (PNCs) of the PVN, which synthesize corticotropin-releasing hormone (CRH) and secrete it into the portal circulation of the anterior pituitary. PNCs receive inputs from cortical, thalamic, hypothalamic, medullary and spinal neuronal sources as well as circulating endocrine signals (reviewed in Ferguson et al., 2008; Levy and Tasker, 2012). Of particular importance for the control and modulation of PNCs activity are glutamatergic and GABAergic inputs, both afferent and local (Busnardo et al., 2013; reviewed in Ziegler and Herman, 2002; Ferguson et al., 2008).

Several studies have demonstrated that with repeated stress, the efficacy of excitatory and inhibitory synapses on PNCs undergoes considerable modifications, which may be related to repeated stress-induced sensitization of the HPA axis (reviewed in Bains et al., 2015; Herman and Tasker, 2016). For example, chronic (2–3 weeks) variable stress results in an increase in the frequency of miniature excitatory postsynaptic currents (mEPSC) in PNCs (Franco et al., 2016) and a decrease in the frequency of miniature inhibitory postsynaptic currents (mIPSC; Verkuyl et al., 2004; Franco et al., 2016). We have previously demonstrated that restraint of rats for 10 min repeated twice daily for 3 days resulted in an increase in the frequency of spontaneous EPSCs (sEPSCs) in PNCs of the PVN with no change in spontaneous inhibitory currents (Kusek et al., 2013). This effect was accompanied by a decrease in the intrinsic membrane excitability of PNCs (Kusek et al., 2013). However, in animals daily exposed to the same stressor for prolonged time periods a progressive reduction of the response of the HPA axis to the homotypic stressor is often observed, indicative of adaptation or habituation to the stimulus (reviewed in Martí and Armario, 1998; Rabasa et al., 2015). In line with these findings, while exposure of rats to restraint repeated for 3 days resulted in an increase in the homotypic stress-induced corticosterone surge at 24 h after the last restraint (Gadek-Michalska et al., 2011, 2013), after repeating the restraint stress over 7 days the response of the HPA axis did not differ from control (Gadek-Michalska et al., 2013). Therefore, in the present study we aimed to assess whether changes in synaptic activity and excitability of PNCs observed 24 h after restraint repeated twice daily for 3 days (Kusek et al., 2013) persist when exposure to restraint stress is repeated over 7 days. Moreover, we compared the effects of repeated restraint with immediate and delayed (24 h) consequences of a single restraint.

In the PVN of rats exposed to stressful conditions increased levels of neuronal nitric oxide synthase (nNOS) mRNA and protein have been observed (reviewed in Orlando et al., 2008; Mancuso et al., 2010). Inducible NOS (iNOS) activity has also been implicated in the regulation of the HPA axis activation (Gadek-Michalska et al., 2012). It has been shown that NOS blockers inhibit immobilization stress-induced activation of c-fos in the PVN (Amir et al., 1997) and attenuate immobilization stress-related anxiety response (Joung et al., 2012). Therefore, in the present study we also aimed to determine whether the effects of restraint stress repeated twice daily for 3 days (Kusek et al., 2013) could be ameliorated by administration of Nω-Nitro-L-arginine (L-NNA), a nonselective inhibitor of constitutive and iNOS.

## Materials and Methods

### Animals, Stress Protocols and L-NNA Administration

Experimental procedures were approved by the Animal Care and Use Committee at the Institute of Pharmacology, Polish Academy of Sciences, and were carried out in accordance with the European Community guidelines and national law. Male Wistar rats weighing approximately 100 g at the beginning of the experiment were housed in groups of 4–5 animals in plastic cages (55 × 35 × 20 cm) on a controlled light/dark cycle (the light on: 7.00–19.00), with free access to standard food and tap water. The rats were restrained in metal tubes (diameter: 55 mm) for 10 min either once or two times a day for 3 or 7 days. Control groups comprised of naïve animals remaining in home cages.

L-NNA was dissolved in sterile physiological saline and injected i.p. 15 min before restraint at a dose of 2 mg/ml/kg. Corresponding controls received only saline.

### Plasma Corticosterone Level Measurement

Rats were restrained twice a day for 3 or 7 days and 24 h after the last restraint they were again exposed to homotypic stress for 10 min and rapidly decapitated. Trunk blood was collected on ice into plastic conical tubes containing 200 μl of a solution of 5 mg/ml EDTA and 500 TIU of aprotinin (Sigma). Plasma was separated by centrifugation in a refrigerated centrifuge within 30 min and stored frozen at −20°C until the time of assay. Control rats were restrained once. The concentration of serum corticosterone was measured using the fluorometric method (Gadek-Michalska et al., 2012).

### Slice Preparation, Whole-Cell Recording and Neurobiotin Immunostaining

**Figure 1:**
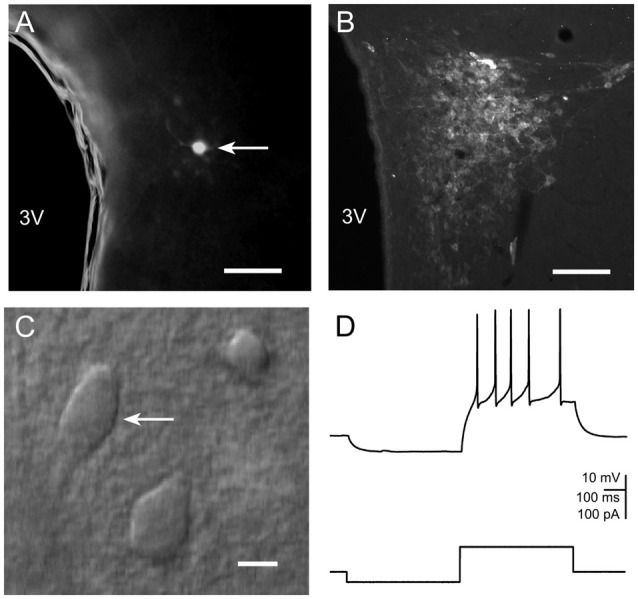
**(A)** Photomicrograph illustrating a neurobiotin-filled presumable parvocellular neurosecretory cell (PNC) in the paraventricular nucleus (PVN). The third ventricle is labeled as 3V. Scale bar: 100 μm. **(B)** Fluorescence image of a PVN slice immunostained for corticotropin-releasing hormone (CRH). Labels as in **(A)**. **(C)** IR-DIC image showing a presumable PVN neurosecretory neuron. Scale bar: 10 μm. **(D)** A representative current clamp recording of a response of the cell shown in **(C)** to current pulses.

Rats were anesthetized with halothane and decapitated between 10:00–11:00 a.m. during the light phase. Animals restrained once were decapitated either immediately after the end of restraint or 24 h later. Rats restrained repeatedly were decapitated 24 h after the last restraint. Brains were rapidly removed and immersed in ice-cold artificial cerebrospinal fluid (ACSF) containing (in mM): NaCl (130), KCl (5), CaCl_2_ (2.5), MgSO_4_ (1.3), KH_2_PO_4_ (1.25), NaHCO_3_ (26) and D-glucose (10), bubbled with a mixture of 95% O_2_ and 5% CO_2_. Brain slices (350 μm) containing the PVN were cut in a coronal plane using a vibrating microtome (VT1000, Leica Microsystems) and subsequently incubated in ACSF at 30°C for at least 3 h. Individual slices were then placed in the recording chamber and superfused at 2.5 ml/min with warm (32°C) modified ACSF in which [NaCl] was raised to 132 mM and [KCl] was lowered to 2 mM. Recordings were performed in the parvocellular neuroendocrine division of the PVN, rich in CRH-immunoreactive cells (Figures 1A,B). Neurons were visualized using the Zeiss Axioskop 2 upright microscope with Nomarski optics, a 40× water immersion lens and an infrared camera (Tokarski et al., 2003). Small-bodied fusiform parvocellular neurons (Figure 1C) were visually distinguished from large-bodied magnocellular neurons (Kania et al., 2017).

Patch pipettes were pulled from borosilicate glass capillaries (Clark Electromedical Instruments) using the Sutter Instrument P-97 puller. The pipette solution contained (in mM): K-gluconate (130), NaCl (5), CaCl_2_ (0.3), MgCl_2_ (2), HEPES (10), Na_2_-ATP (5), Na-GTP (0.4) and EGTA (1). Osmolarity and pH were adjusted to 280 mOsm and 7.2 respectively. Pipettes had an open tip resistance of  ~6 MΩ. In some experiments neurobiotin (0.2%, Vector Laboratories) was included in the pipette solution. Signals were recorded using the Multiclamp 700B amplifier, filtered at 2 kHz and digitized at 20 kHz with the Digidata 1400A interface and Clampex 10 software (Molecular Devices).

Following the experiments where neurobiotin was included in the pipette solution, slices were fixed for 24 h in 4% formaldehyde in PBS. After several washings, the tissue was incubated in phosphate-buffered saline containing 0.6% Triton X-100 (Sigma-Aldrich) for 24 h and subsequently with 0.3% Triton X-100 and Cy3-conjugated ExtrAvidin (1:200; Sigma-Aldrich) for 48 h. Slices were washed and mounted on glass slides, coverslipped with Vectashield (Vector Laboratories), and examined using Leica DM 6000 B microscope equipped with a digital camera (MBF CX9000, Williston, VT USA; Figure 1A).

### Analysis of Intrinsic Excitability and Miniature Excitatory and Inhibitory Postsynaptic Currents

Response characteristics of the recorded neurons were evaluated in current clamp mode. PVN neuroendocrine parvocellular neurons can be distinguished from non-neurosecretory parvocellular cells and from magnocellular neurons based on the lack of transient outward rectification and low-threshold spike (Luther et al., 2002). Only cells showing the firing pattern characteristic of neuroendocrine PNCs (Figure 1D) and exhibiting no spontaneous spiking activity at the resting membrane potential were subjected to analysis.

To determine the relationship between the injected current and the firing rate, the membrane potential was adjusted to −57 mV by current injection. Then, neurons were hyperpolarized with rectangular current pulses (−60 pA) and then depolarized by current steps of increasing amplitude (increment: 20 pA; Luther et al., 2002). The number of action potentials evoked by respective current pulses was plotted and a linear regression line was fitted to the data. Gain (slope) and firing threshold (current extrapolated at a zero firing rate) were determined from the fitted regression line (Bekisz et al., 2010). After characterizing the membrane excitability tetrodotoxin (TTX, 500 nM, Tocris) was added to the ACSF and neurons were voltage-clamped at −76 mV. mEPSCs were recorded for 4 min after which cells were voltage-clamped at 0 mV to record mIPSCs for 4 min as described previously (Kusek et al., 2013). We have previously shown that inward currents recorded from PNCs of the PVN at −76 mV were completely blocked by kynurenic acid and outward currents recorded at 0 mV were completely blocked by picrotoxin (Kusek et al., 2013). Recordings were analyzed off-line using the Mini Analysis software (Synaptosoft). Data were accepted for analysis when access resistance was below 18 MΩ and remained stable during recordings.

### CRH Immunostaining

Animals were anesthetized and transcardially perfused with saline followed by 4% paraformaldehyde in 0.1 M PBS (pH 7.4). Brains were removed, postfixed in 4% paraformaldehyde in PBS for 24 h at 4°C and sectioned on a vibrating microtome (VT1000S, Leica) into 50 μm thick coronal sections (stereotaxic coordinates: dorsoventral −8 mm, lateromedial ±0.4 mm, and anteroposterior −1.88 mm from bregma). Free-floating sections were washed in PBS and incubated in 5% normal donkey serum (NDS, Sigma-Aldrich) and 0.1% Triton X-100 (Sigma-Aldrich) in PBS for 1 h. They were then incubated (48 h, 4°C) in PBS containing goat anti-CRH antibodies (1:100; Santa Cruz Biotechnology: sc-1759). After several washes the sections were incubated in PBS with Alexa Fluor 488-conjugated donkey anti-goat antibodies (1:200, 2 h, Thermo Fisher Scientific). Finally, the sections were mounted onto gelatin-coated slides and coverslipped using PBS-buffered glycerol. The sections were analyzed and photographed using a Leica DM 6000 B microscope equipped with a digital camera (MBF CX9000, Williston, VT USA; Figure 1B).

### Statistical Analysis

After testing for normality (Shapiro-Wilk), statistical analysis of electrophysiological data was carried out using Student’s *T*-test (SigmaPlot 12, Systat Software). The Kolgomorow-Smirnov test was used to compare cumulative distributions of synaptic currents (MiniAnalysis, Synaptosoft). Corticosterone concentration was evaluated using the one-way analysis of variance (ANOVA) followed by individual comparisons with Duncan’s *post hoc* tests (Prism, Graphpad). Data are presented as mean ±SEM.

## Results

### Restraint Repeated Over 3 Days Induces Sensitization of the HPA Axis, Changes in mEPSC Characteristics and Decreases Excitability of PNCs for Longer than 24 h

In the blood plasma of rats exposed to 10 min restraint 24 h after the last one of six restraint sessions repeated over 3 days, plasma corticosterone levels increased by a larger amount than in animals which were restrained only once (145.8 ± 3.75%, *n* = 10 and *n* = 11, *f* = 11.515, df = 20 *p* = 0.003) indicating a sensitization of the reaction of the HPA axis to homotypic stress.

**Figure 2:**
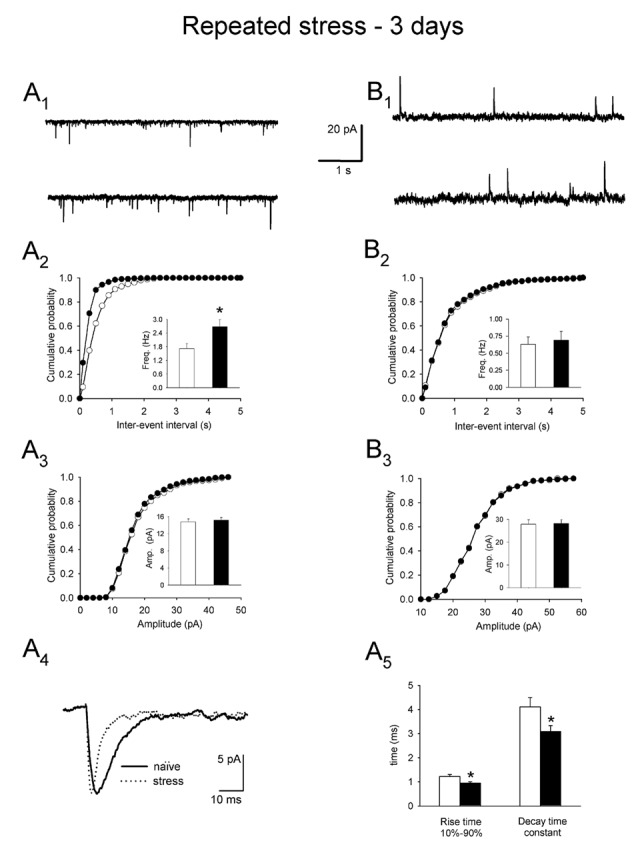
**Miniature postsynaptic currents and plasma corticosterone levels after restraint repeated over 3 days followed by a 24 h delay before measurements. (A_1_)** Representative raw recording of miniature excitatory postsynaptic currents (mEPSCs) in control preparation (*upper trace*) and in slice obtained after restraint (*lower trace*). **(A_2_,A_3_)** Cumulative probability plots of inter-event interval and amplitude distributions in control (*white circles/bars*) and stress (*black circles/bars*) conditions. *Insets*: comparison of the average values (±SEM). **(A_4_)** Overlaid average mEPSCs recorded from representative PNCs from a control rat (*naïve*) and a rat subjected to repeated restraint (*stress*). **(A_5_)** Comparison of the average (±SEM) rise time and decay time constant of mEPSCs in control (*white bars*) and stress (*black bars*) conditions. **(B_1_)** Representative raw recording of mIPSCs in control (*upper trace*) and after restraint (*lower trace*). **(B_2_,B_3_)** Cumulative probability plots of inter-event interval and amplitude distributions in control (*white circles/bars*) and stress (*black circles/bars*) conditions. *Insets*: comparison of the average values ±SEM. *Indicates *p* < 0.05.

The mean mEPSC frequency in PNCs of rats restrained six times over 3 days, 24 h after the last restraint, was higher than in control cells (2.68 ± 0.31 Hz vs. 1.72 ± 0.22 Hz, respectively; *n* = 16 and 11, *t* = 0.532, df = 25, *p* = 0.012; Figures 2A_1,2_). Moreover, the mean rise time of mEPSCs was shorter (0.95 ± 0.05 ms vs. 1.22 ± 0.09 ms, *n* = 16 and 11, *t* = −1.783, df = 25, *p* = 0.015, Figures 2A_4,5_), as was the decay time constant (3.09 ± 0.24 ms vs. 4.11 ± 0.39 ms, *n* = 16 and 11, *t* = −1.879, df = 25, *p* = 0.029, Figures 2A_4,5_) in the experimental group compared to the control group. Repeated restraint stress did not affect the mean amplitude of mEPSCs (15.20 ± 0.59 pA vs. 14.80 ± 0.65 ms, *n* = 16 and 11, *t* = 0.327, df = 25, *p* = 0.681; Figure 2A_3_).

In contrast to mEPSCs, no effects of restraint stress repeated over 3 days have been observed on mIPSC characteristics in comparison to the control group. The mean frequency (0.69 ± 0.13 Hz vs. 0.63 ± 0.11 Hz, *n* = 19 and 10, respectively; *t* = −0.198, df = 27, *p* = 0.732; Figures 2B_1,2_) and the mean amplitude (29.12 ± 1.68 pA vs. 27.86 ± 1.89 pA, *t* = 0.321, df = 27, *p* = 0.581; Figure 2B_3_), as well as the rise time (1.34 ± 0.09 ms vs.1.32 ± 0.10 ms, df = 27, *p* = 0.316) and the decay time constant (9.78 ± 0.90 vs. 9.71 ± 0.80 ms, *t* = 1.986, df = 27, *p* = 0.321) of mIPSCs were similar in both groups.

Analyses of the relationship between the injected current and PNC firing rate in slices prepared 24 h after the last of six restraint sessions revealed a decrease in the mean gain in the stressed vs. control group (0.13 ± 0.01 Hz/pA vs. 0.17 ± 0.02 Hz/pA, *n* = 29 and 19, respectively; *t* = 1.213, df = 46, *p* = 0.026; Table 1), confirming previous findings (Kusek et al., 2013). There were no significant differences between PNCs originating from stressed and control animals in either the resting membrane potential or the input resistance (Table 1).

**Table 1:** **Effects of restraint stress on basic electrophysiological characteristics and excitability of parvocellular neurosecretory cells (PNCs)**.

	Acute stress—early effects		Acute stress—after 24 h		Repeated stress—3 days		Repeated stress—7 days		Repeated stress—3 days	
	Stress	Control	Stress	Control	Stress	Control	Stress	Control	+NaCl	+L-NNA
Resting membrane potential [mV]	58.87 ± 0.28	59.12 ± 0.29	58.78 ± 0.25	58.33 ± 0.26	58.40 ± 0.34	58.72 ± 0.33	58.98 ± 0.31	59.08 ± 0.29	59.31 ± 0.38	59.64 ± 0.39
Input resistance [MΩ]	521 ± 13.00	512 ± 11.00	501 ± 15.00	489 ± 14.00	584 ± 21.00	546 ± 14.00	511 ± 14.00	516 ± 16.00	579 ± 20.00	599 ± 19.00
Threshold [pA]	54.89 ± 4.81	55.14 ± 3.10	54.87 ± 3.12	54.55 ± 2.90	54.64 ± 5.24	54.11 ± 3.20	54.98 ± 2.32	55.12 ± 2.00	54.94 ± 2.89	55.01 ± 2.94
Gain [Hz/pA]	0.16 ± 0.01	0.17 ± 0.01	0.16 ± 0.01	0.16 ± 0.01	0.13 ± 0.01*	0.17 ± 0.02	0.16 ± 0.01	0.16 ± 0.01	0.12 ± 0.02	0.13 ± 0.01
Number of rats/cells	9/25	8/23	8/19	7/16	8/29	6/19	7/21	6/15	4/8	4/9

*Indicates p value < 0.05.

### Effects of Acute Restraint Stress on mEPSCs and mIPSCs Last Less than 24 h

**Figure 3:**
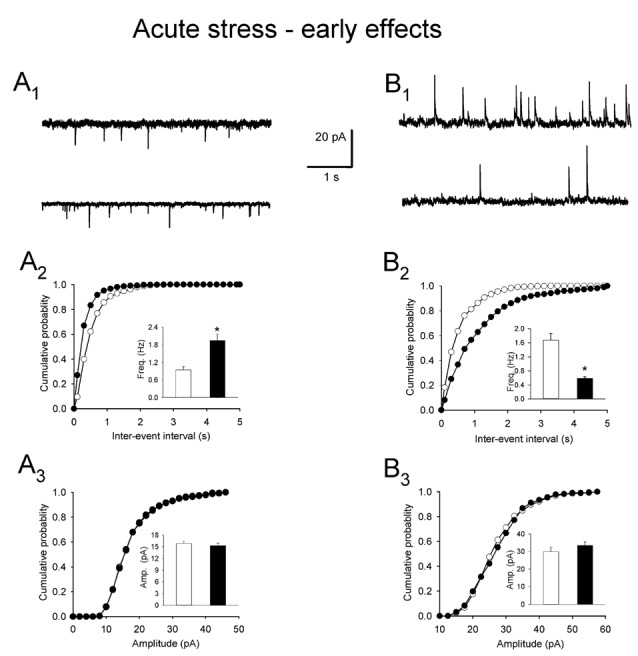
**Effects of acute restraint stress on miniature postsynaptic currents. (A_1_)** Representative raw recording of mEPSCs from PNCS in control preparation (*upper trace*) and after acute restraint (*lower trace*). **(A_2_,A_3_)** Cumulative probability plots of inter-event interval and amplitude distributions in control (*white circles/bars*) and acute stress (*black circles/bars*) conditions. *Insets*: comparison of average values ±SEM. **(B_1_)** Representative recording of miniature inhibitory postsynaptic currents (mIPSCs) in control conditions (*upper trace*) and after acute restraint (*lower trace*). **(B_2_,B_3_)** Cumulative probability plots of inter-event interval and amplitude distributions in control (*white circles/bars*) and acute stress (*black circles/bars*) conditions. *Insets*: comparison of the average values (±SEM). *Indicates *p* < 0.05.

In order to compare the effects of restraint repeated six times over 3 days with acute stress-related changes in mEPSCs, mIPSCs and excitability of PNCs, a set of slices were prepared immediately after the end of a 10 min restraint. In these preparations, the mean frequency of mEPSCs was markedly higher than in cells originating from control rats (1.95 ± 0.22 vs. 0.99 ± 0.10 Hz, *n* = 14 and 9, respectively; *t* = −3.95, df = 21, *p* = 0.0009; Figures 3A_1,2_). The mean amplitude of mEPSCs did not differ between cells from stressed and control rats (15.13 ± 0.58 vs. 15.08 ± 0.54 pA, respectively; *t* = −1.482, df = 21, *p* = 0.15; Figure 3A_3_). The rise time (0.99 ± 0.03 vs. 1.08 ± 0.03 ms, *t* = 0.833, df = 21, *p* = 0.412) and the decay time constant of mEPSCs (3.89 ± 0.21 vs. 4.07 ± 0.15 ms, *t* = 1.216, df = 21, *p* = 0.234) were also similar in both groups. In PNCs obtained from stressed rats, the mean frequency of mIPSCs was markedly lower than in control neurons (0.58 ± 0.05 vs. 1.68 ± 0.18 Hz, respectively; *t* = 6.847, df = 30, *p* = 0.0011; Figures 3B_1,2_), but stress affected neither the mean amplitude (33.51 ± 2.03 vs. 29.19 ± 2.40 pA, *t* = −1.328, df = 30, *p* = 0.194; Figure 3B_3_), the rise time (1.09 ± 0.04 vs. 1.09 ± 0.03 ms, *t* = −0.568, df = 30, *p* = 0.727) nor the decay time constant (8.07 ± 0.25 vs. 7.76 ± 0.29 ms, *t* = −0.787, df = 30, *p* = 0.438) of mIPSCs. Acute stress also did not influence the excitability of PNCs (Table 1).

**Figure 4:**
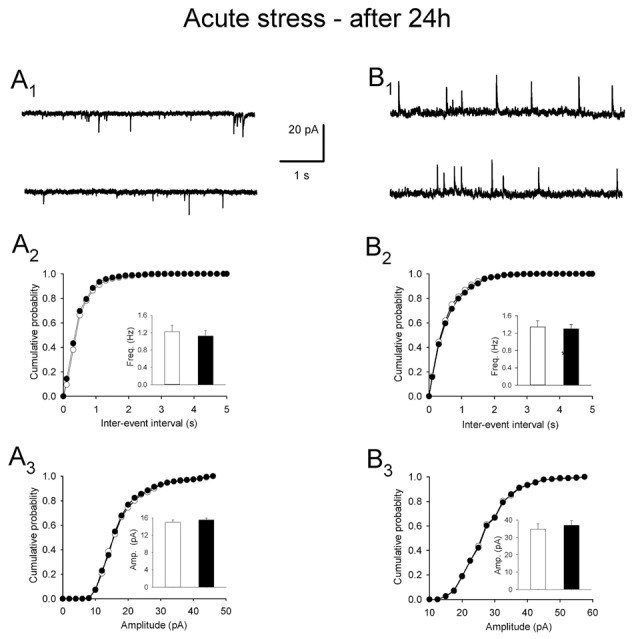
**Miniature postsynaptic currents after acute restraint stress followed by a 24-h delay before recordings. (A_1_)** Representative raw recording of mEPSCs in control conditions (*upper trace*) and after restraint (*lower trace*). **(A_2_,A_3_)** Cumulative probability plots of inter-event interval and amplitude distributions in control (*white circles/bars*) and stress (*black circles/bars*) conditions. *Insets*: comparison of the average values (±SEM). **(B_1_)** Representative recording of mIPSCs in control (*upper trace*) and after restraint (*lower trace*). **(B_2_,B_3_)** Cumulative probability plots comparing inter-event interval and amplitude distributions in control (*white circles/bars*) and stress (*black circles/bars*) conditions. *Insets*: comparison of the average values ±SEM.

To test the longevity of acute stress-related changes in mEPSCs and mIPSCs in PNCs, another set of slices were prepared 24 h after a single 10 min restraint. In these preparations, acute stress-induced effects were no longer present. The difference between the mean frequency of mEPSCs in cells from stressed and control animals was not significant (1.22 ± 0.15 vs. 1.13 ± 0.12 Hz, *n* = 12 and 8, respectively; *t* = 2.878, df = 18, *p* = 0.712; Figures 4A_1,2_). Similarly, there was no difference in the mean frequency of mIPSC between both groups (1.30 ± 0.10 vs. 1.34 ± 0.15 Hz, *n* = 20 and 12, *t* = 6.847, df = 30, *p* = 0.157; Figures 4B_1,2_). Other parameters characterizing mEPSCs: mean amplitude (15.51 ± 0.42 vs. 14.98 ± 0.53 pA, *t* = 0.325, df = 18, *p* = 0.19; Figure 4A_3_), rise time (1.14 ± 0.03 vs. 1.03 ± 0.05 ms, *t* = 1.435, df = 18, *p* = 0.534) and decay time constant (3.68 ± 0.21 vs. 3.82 ± 0.14 ms, *t* = 0.829, df = 18, *p* = 0.324) were not different as were not the parameters characterizing mIPSCs: mean amplitude (36.99 ± 2.76 pA vs. 34.86 ± 3.09 pA, *t* = 2.988, df = 30, *p* = 0.321; Figure 4B_3_), rise time (1.09 ± 0.04 ms vs. 1.12 ± 0.03 ms, *n* = 20 and 12, *t* = 2.978, df = 30, *p* = 0.514) and decay time constant (8.47 ± 0.22 vs. 8.52 ± 0.31, *n* = 20 and 12, *t* = −0.987, df = 30, *p* = 0.142). There was no difference in the excitability of PNCs between the experimental and the control group (Table 1). Thus, in contrast to the effects of restraint repeated over 3 days, the effects of a single restraint faded in less than 24 h.

### No Changes in the Activation of the HPA Axis and mEPSC Characteristics, and Excitability of PNCs Are Evident after Restraint Repeated Over 7 days

There were no differences in corticosterone plasma levels between rats subjected to a single acute restraint session after having restraint sessions twice daily for 7 days and animals which were restrained only once (99 ± 10.69% *n* = 7 and *n* = 6, respectively, *f* = 0.00246, df = 12, *p* = 0.96).

**Figure 5:**
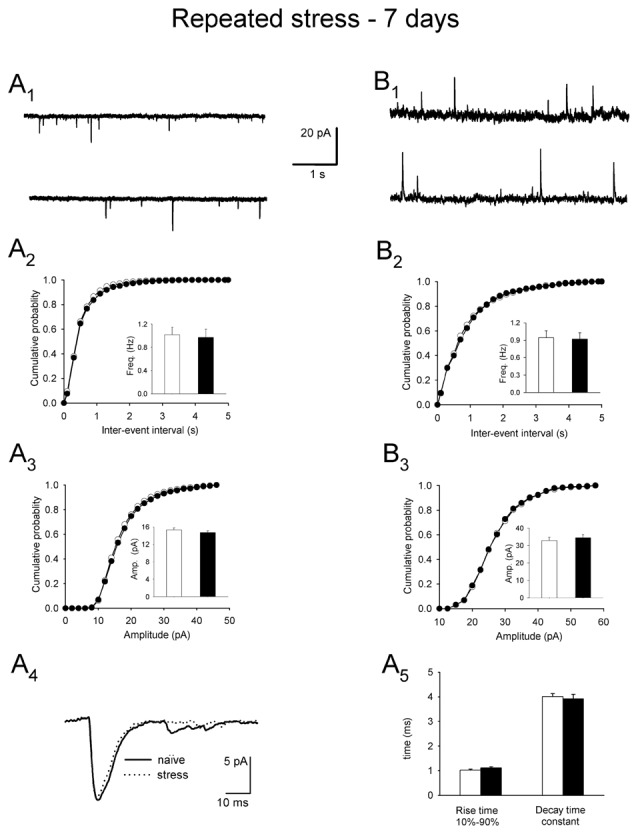
**Miniature synaptic currents and plasma corticosterone levels after restraint repeated over 7 days followed by a 24 h delay before recordings. (A_1_)** Representative raw recording of mEPSCs in control (*upper trace*) and after restraint (*lower trace*). **(A_2_,A_3_)** Cumulative probability plots of inter-event interval and amplitude distributions in control (*white circles/bars*) and stress (*black circles/bars*) conditions. *Insets*: comparison of the average values ±SEM. **(A_4_)** Overlaid average mEPSCs recorded from representative PNCs from a control rat (*naïve*) and a rat subjected to repeated restraint (*stress*). **(A_5_)** Comparison of the average (±SEM) rise time and decay time constant of mEPSCs in control (*white bars*) and stress (*black bars*) conditions. **(B_1_)** Representative recording of mIPSCs in control conditions (*upper trace*) and after restraint (*lower trace*). **(B_2_,B_3_)** Cumulative probability plots of inter-event interval and amplitude distributions in control (*white circles/bars*) and stress (*black circles/bars*) conditions. *Insets*: comparison of the average values ±SEM.

There were no significant differences between PNCs originating from animals stressed 14 times over 7 days and controls in the resting membrane potential, input resistance and excitability of PNCs (Table 1). Restraint repeated over 7 days had no effect on parameters characterizing mEPSCs: mean frequency (0.97 ± 0.13 vs. 1.01 ± 0.14, *n* = 14 and 9, the experimental and control group, respectively; *t* = 2.121, df = 21, *p* = 0.198; Figures 5A_1,2_), mean amplitude (15.29 ± 0.50 vs. 14.71 ± 0.42 pA, *t* = 0.125, df = 21, *p* = 0.224; Figure 5A_3_), rise time (1.11 ± 0.05 vs. 1.02 ± 0.04 ms, *t* = 0.875, df = 21, *p* = 0.461; Figures 5A_4,5_) and decay time constant (3.91 ± 0.19 vs. 4.01 ± 0.12 ms, *t* = 0.829, df = 21, *p* = 0.324; Figures 5A_4,5_).

There were also no changes in the mean frequency of mIPSCs (0.92 ± 0.11 vs. 0.94 ± 0.12 Hz, *n* = 11 and 9, the experimental and control group, respectively; *t* = 6.847, df = 18, *p* = 0.157; Figures 5B_1,2_), mean amplitude (34.51 ± 1.68 vs. 32.86 ± 1.89 pA, *t* = 1.452, df = 18, *p* = 0.199; Figure 5B_3_), as well as the rise time (1.11 ± 0.03 vs. 1.23 ± 0.03 ms, *n* = 11 and 9, *t* = 1.656, df = 18, *p* = 0.093) and the decay time constant (8.47 ± 0.22 vs.8.52 ± 0.31, *n* = 11 and 9, *t* = 0.563, df = 18, *p* = 0.665). Thus, in contrast to restraint repeated over 3 days, there were no changes in mEPSC/mIPSC characteristics and PNC excitability in rats after restraint repeated over 7 days.

### Effects of Restraint Repeated Six Times Over 3 Days on the Characteristics of mEPSCs Are Attenuated by Treatment of Rats with a NOS Blocker

To test whether the effects observed 24 h after the last of six restraint sessions repeated over 3 days could be related to the activity of NOS, its blocker L-NNA was administered 15 min before each restraint.

The relationship between injected current and firing rate showed no difference in the mean gain of PNCs in slices prepared 24 h after the last of six restraints from rats receiving L-NNA vs. rats receiving NaCl (0.13 ± 0.01 Hz/pA vs. 0.12 ± 0.02 Hz/pA, *n* = 9 and 8, respectively; *t* = −0.236, df = 16, *p* = 0.435; Table 1). Moreover, L-NNA treatment had no effect on the mean gain of PNCs in slices prepared from animals not subjected to any restraint sessions (0.17 ± 0.02 Hz/pA (L-NNA) vs. 0.16 ± 0.01 Hz/pA (NaCl), *n* = 8 and *n* = 8 respectively, *t* = 0.154, df = 15, *p* = 0.243). Thus, L-NNA did not ameliorate the influence of repeated restraint on the excitability of PNCs. There were no significant differences between PNCs originating from both groups of animals in the resting membrane potential, input resistance and threshold values.

**Figure 6:**
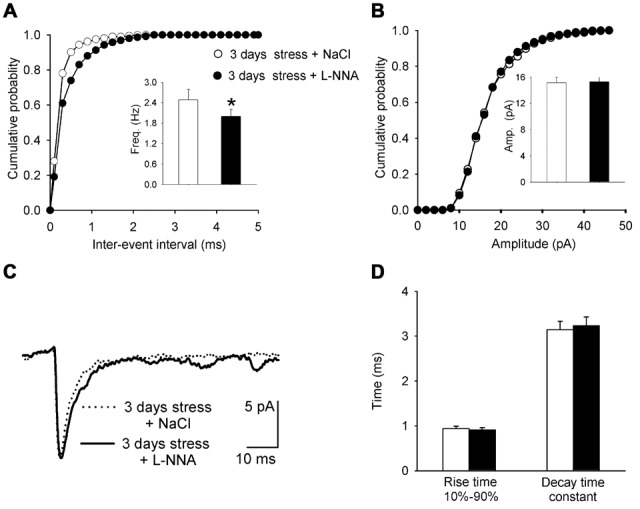
**Miniature synaptic currents after restraint repeated over 3 days followed by a 24-h delay before recordings from rats receiving NΩ-Nitro-L-arginine (L-NNA) before each restraint session. (A,B)** Cumulative probability plots of inter-event interval and amplitude distributions from stressed rats receiving NaCl (*white circles/bars*) and L-NNA (*black circles/bars*). **(C)** Overlaid average mEPSCs recorded from a control rat receiving NaCl and a rat treated with L-NNA. **(D)** Comparison of the average (±SEM) rise time and decay time constant of mEPSCs in PNCs from stressed rats receiving NaCl (*white bars*) and those receiving L-NNA (*black bars*). *Indicates *p* < 0.05.

In slices prepared 24 h after the last of six restraint sessions, the mean mEPSC frequency in PNCs from rats subjected to restraint and receiving L-NNA was significantly lower than in PNCs from rats subjected to restraint and receiving saline (1.99 ± 0.192 Hz vs. 2.48 ± 0.234 Hz, respectively; *n* = 9 and 8, *t* = 1.510, df = 16, *p* = 0.0238; Figure 6A). Thus, treatment of stressed rats with L-NNA resulted in a shift of the mean mEPSC frequency towards the level observed in PNCs of control, unstressed animals (1.99 ± 0.192 Hz vs. 1.72 ± 0.221 Hz, respectively; *n* = 9 and 11, *t* = 0.404, df = 19, *p* = 0.192). However, the mEPSC rise time and the decay time constant were not different between PNCs in the stressed group receiving L-NNA vs. stressed group receiving NaCl (rise time: 0.91 ± 0.049 ms vs. 0.94 ± 0.052 ms, respectively; *n* = 9 and 8, *t* = −0.223, df = 16, *p* = 0.254; decay time constant: 3.23 ± 0.201 ms vs. 3.14 ± 0.198 ms, respectively; *n* = 9 and 8, *t* = 1.783, df = 16, *p* = 0.199, Figures 6C,D). Similarily, L-NNA treatment had no effect on the mEPSC rise time and the decay time constant in slices prepared from animals not subjected to any restraint sessions (rise time: 1.17 ± 0.0512 ms vs. 1.09 ± 0.0496 ms, respectively; *n* = 8 and 8, *t* = −1.3264, df = 15, *p* = 0.341; decay time constant: 3.99 ± 0.154 ms vs. 4.11 ± 0.168 ms, respectively; *n* = 8 and 8, *t* = 0.6578, df = 15, *p* = 0.212). There were also no differences in the mean mEPSC amplitude (15.31 ± 1.121 pA vs. 15.14 ± 0. 894 pA, respectively; *n* = 9 and 8, *t* = 0.138, df = 16, 0.768, Figure 6B). Thus, L-NNA did not ameliorate the influence of repeated restraint on kinetic parameters characterizing mEPSCs. Repeated administration of L-NNA alone did not exert influence on the mean frequency and amplitude of mEPSCs (data not shown).

## Discussion

The present study demonstrates an increase in the mean frequency and changes in the kinetics of mEPSCs as well as a decrease in the excitability of PNCs in rats that were restrained six times over 3 days, which confirms and extends our earlier work employing recordings of sEPSCs (Kusek et al., 2013). However, no such changes were observed in slices prepared from animals restrained repeatedly for 7 days twice a day. While restraint repeated six times over 3 days resulted in an increase in corticosterone release by homotypic stress, no such effect was evident after restraint repeated 14 times over 7 days, confirming earlier results (Gadek-Michalska et al., 2011, 2013). These findings are consistent with the initial sensitization of the HPA axis reactivity by restraint repeated over 3 days, followed by adaptation when animals were subjected to repeated restraint over a prolonged time period (7 days).

The increase in the mean mEPSC frequency found in the present study is likely to result from an increased probability of spike-independent release of neurotransmitter quanta from presynaptic terminals (Franco et al., 2016). It is also possible that the mechanism underlying these effects involves an increase in the number of presynaptic release sites. It has been shown that repeated variable stress increases the number of VGLUT2-immunopositive terminals and asymmetrical (glutamatergic) synapses on CRH neurons, however, these effects occurred after longer-lasting exposure to stress (Flak et al., 2009; Miklós and Kovács, 2012). Both possibilities point to a presynaptic mechanism of the observed effect on the frequency of mEPSCs which lasts for at least 1 day after the last restraint. Spike-independent neurotransmitter release has been shown to have functional significance as a key regulator of homeostatic synaptic plasticity (reviewed in Kavalali, 2015).

The observed shortening of the rise time and the decay time constant of mEPSCs are likely to reflect changes in activation and deactivation kinetics of postsynaptic AMPA receptors (Marty et al., 2011). To our knowledge stress-induced changes in AMPA receptor subunit composition, which might explain observed changes, have not been reported in PNCs of the PVN yet, however it has been shown that immobilization stress results in an increased level of GluN1 mRNA in the hippocampus and in the PVN and a decrease in GluA1 mRNA in the hippocampus (Bartanusz et al., 1995). Our data also demonstrate that the membrane excitability of recorded cells remains decreased when tested 1 day after the end of repeated restraint sessions. Neurons obtained from stressed rats generated fewer action potentials compared to control cells, however neither the resting membrane potential nor the input resistance were altered. This effect might be related to enhanced current flow through large conductance Ca^2+^-activated K^+^(BK) channels (Bekisz et al., 2010), which are known to modulate the activity of PVN neurosecretory neurons (Brunton et al., 2007). It is tempting to speculate that the reduced excitability of PVN PNCs represents a homeostatic compensatory mechanism acting in response to increased spontaneous excitatory synaptic input to PNCs. No effects of acute stress on excitatory transmission and membrane excitability were detectable in PNCs in slices prepared 24 h after the end of single restraint. Thus, the effects observed 24 h after restraint repeated over 3 days may be interpreted in terms of stress-induced plasticity.

In slices prepared immediately after single restraint, we observed a decrease in the frequency of mIPSCs. These data are consistent with the results of Verkuyl et al. (2005) who reported a decrease in the mean frequency of mIPSCs in PNCs following a single 1 h restraint session, due to a decrease in GABA release probability. Hewitt et al. (2009) have shown that acute restraint stress induces a depolarizing shift in the reversal potential of GABA_A_-mediated synaptic currents in PNCs due to downregulation of the transmembrane anion transporter KCC2, which results in lower IPSC amplitudes. We have not observed a decrease in mIPSC amplitudes, possibly due to differences in the experimental design regarding the duration of restraint. In slices prepared just after restraint we have also observed an increase in the frequency of mEPSCs, which has not been reported previously. Available data indicate that glucocorticoids induce a rapid presynaptic inhibition of glutamate release via endocannabinoids acting as retrograde messengers (Di et al., 2003). Another study reported no change in the frequency of sEPSCs after acute immobilization lasting 30 min (Kuzmiski et al., 2010). A likely reason for the increase in mEPSC frequency observed in our experiments is again the difference in the experimental design including shorter (10 min) immobilization and a longer delay (3–4 h) before the time of slice preparation and the recording. The latter study, however, also demonstrated that acute immobilization results in an increase in the release probability of glutamate, which is unmasked following a burst of high-frequency afferent activity (Kuzmiski et al., 2010). This result may be of relevance to our observations. It should also be noted that noradrenergic inputs to the PVN modulate glutamate and GABA release within this nucleus (reviewed in Levy and Tasker, 2012). Nevertheless, the immediate effects of acute restraint stress that we observed lasted less than 24 h. After a single restraint session lasting 10 min, plasma cortisol levels return to baseline values within 1 h (Gadek-Michalska et al., 2013) and the effects of corticosterone on neuronal excitability, investigated in other brain structures, decline to baseline values within hours (Joëls, 2011).

No changes in synaptic activity and neuronal excitability were observed in PNCs in slices prepared from animals subjected to 14 restraint sessions over 7 days. This result is consistent with the observation that after an identical treatment of the animals the homotypic stress-induced corticosterone surge is not different from control (this study and Gadek-Michalska et al., 2013). Repeated restraint has been shown to lead to a systems-level adaptation producing a widespread attenuation of the neural response to restraint evident in brain structures involved in processing of sensory information (Girotti et al., 2006), however, the mechanisms of these processes remain to be elucidated.

Obtained results indicate that administration of an unspecific NOS blocker prevented the increase in mEPSC frequency, however in PNCs from animals receiving L-NNA and subjected to restraint both the kinetics of mEPSCs and the membrane excitability were altered to a similar extent as in stressed rats not receiving L-NNA. Thus, it appears that L-NNA treatment selectively interfered with stress-induced presynaptic but not postsynaptic effects. NOS is present in the PVN, median eminence and the pituitary, which suggests that NO plays a physiological role in regulating the secretion of CRH and other hypothalamic peptides as well as ACTH (Lee et al., 1999; reviewed in Mancuso et al., 2010). NO is thought to modulate synaptic transmission through the activation of guanylate cyclase (GC), leading to enhanced synthesis of cyclic guanosine monophosphate (cGMP), which regulates neurotransmitter release (reviewed in Esplugues, 2002; Mancuso et al., 2010). Acute immobilization stress has been shown to result in Fos expression and expression of iNOS with no changes in nNOS level in PVN neurons (Yamaguchi et al., 2010). Restraint stress repeated over 3 days has been shown to result in an increase in the level of iNOS but not nNOS protein level in the hypothalamus (Gadek-Michalska et al., 2012). Thus, it is conceivable that the increase in mEPSC frequency observed after stress in our study depends on the activity of iNOS in the PVN, however, it should be noted that systemically administrated NOS inhibitor exerts its effects also in other brain structures (Gadek-Michalska et al., 2012) and possibly in other organs. The mechanisms of the remaining processes remain to be elucidated.

## Author Contributions

GH, KT and MK conceived and designed the experiments. MK, AT, AG-M, BS and KT performed the experiments. MK, AT, MS, KT and GH analyzed and interpreted the obtained data. MK, AT, KT and GH wrote the article. MS helped with the manuscript preparation. MK, AT, MS, AG-M, BS, KT and GH granted a final approval of the version of the article to be published and agreed to be accountable for all aspects of the work.

## Funding

The study was supported by Ministry of Science and Higher Education (Warsaw, Poland) grant no 2012/07/N/NZ4/02687, and by statutory funds from the Institute of Pharmacology, Polish Academy of Sciences, Krakow, Poland. MK and MS are beneficiaries of the KNOW PhD scholarship sponsored by the Ministry of Science and Higher Education, Poland.

## Conflict of Interest Statement

The authors declare that the research was conducted in the absence of any commercial or financial relationships that could be construed as a potential conflict of interest.
